# Evaluation of surface topography of two different heat treated NiTi file systems used with sodium hypochlorite: A stereomicroscope and SEM study

**DOI:** 10.6026/973206300211257

**Published:** 2025-05-31

**Authors:** Anisha Parmar, Niraj Kinariwala, Nirav Parekh, Vishwesh Joshi, Riya Modi, Urooj Desai

**Affiliations:** 1 Department of Conservative Dentistry and Endodontics, Karnavati School of Dentistry, Karnavati University, Gandhinagar, Gujarat, India; 2 Clinical Practice Owner, Smile Rite Dental Clinic, CT, USA; 3 Intern, Karnavati School Of Dentistry, Karnavati University, Gandhinagar, Gujarat, India

**Keywords:** NiTi file, sodium hypochlorite, scanning electron microscope (SEM)

## Abstract

The surface grooves on endodontic files act as stress concentration and it lowers their susceptibility to fatigue fracture. Hence, 80 first mandibular molars were prepared with 40 rotary instruments, 20 One Curve (OC; Micro-Mega) and 20 TruNatomy (TN; Dentsply Sirona,) rotary files. The files were rotated in mesial curved canals with irrigation with 5.25% sodium hypochlorite (Normal temperature and preheated) and inspected under SEM for surface roughness. Stereomicroscope analysis was done for Unwinding and Distortion. One Curve files showed signs of deformation and distortion earlier as compared to TruNatomy in curved canals. Sodium hypochlorite (Preheated form) affects the surface topography and fatigue resistance of the files.

## Background:

The introduction of NiTi rotary tools in endodontic treatment, has improved operator effectiveness, maintained canal form and rendered root canal instrumentation safe [1]. The main issue with using NiTi rotary instruments is the unexpected separation of the instrument during a clinical operation. The several studies have shown that the existence of grooves mostly on surface of the metal may reduce the number of cycles before endodontic files fatigue fracture. These grooves onto endodontic files may also operate as stress concentration locations, lowering their susceptibility to fatigue fracture, according to certain research [2]. The newest NiTi endodontic file variants have been launched, including CM files, M-wire technology files and R phase wire technology, which are more flexible and fatigue-resistant [3]. The One Curve (OC; Micro Mega), a rotary NiTi system has just entered the market, reduces the duration of instrumentation for root canals while preserving their original form. The root canal may be easily accessed because to the controlled memory (CM) quality of the C-wire heat-treated NiTi material used in the OC system, which allows for flexible modification of the file's form before entering the canal [4]. A recently introduced file system termed TruNatomy (TN; Dentsply Sirona, Maillefer, Ballaigues, Switzerland) was constructed from 0.8 mm NiTi wire rather than the 1.2 mm NiTi wire used to make most generic files, followed by a specific heat treatment. According to the company, heat treatment and file design work together to provide more flexibility and effective shaping while just removing dentin when required clinically [2, 3-4]. Sodium hypochlorite is one of the aforementioned compounds that are utilized most frequently. It has been established that the extremely alkaline solution of NaOCl causes detectable corrosion at concentrations of 5-5.25%. Files used with NaOCl causes micro pitting because it preferentially eliminates nickel from the instrument's surface, which has a detrimental effect on the physical and mechanical characteristics of NiTi files [5, 6]. Unfortunately, only a few researches have examined the impact of NaOCl on the surface of NiTi instruments that have undergone heat treatment [7]. Therefore, it of interest to compare the surface topographies of two different NiTi files, developed with different wire technologies, when used along with sodium hypochlorite (Normal and Preheated Form).

## Methodology:

The 80 complete, fully matured first mandibular molars that were removed because of poor periodontal health were the subject of the study. All of the samples were radiographed to assess the canals and looked at under a dental operating microscope to check for cracks. The teeth with mesial canals having curvature of 20°C-30°C are taken for the study. After being soaked in 10% formalin solution for no more than two weeks following extraction, all the teeth were cleaned using ultrasonic scalers and preserved in 0.9% saline solution until usage after being maintained in 5.25 % NaOCl for two hours. Using the t-test option in the G*Power 3.1 programme (Heinrich Heine University, Dusseldorf, Germany), sample size was calculated, determining two dependent means apart (matched pairs) and the beta power of 0.95 and the alpha-type error of 0.05. The analysis revealed that a minimum of 10 equipment must be included in each group's sample size. A total of 40 rotary instruments, 20 One Curve (OC; Micro-Mega (Besançon, France) and 20 TruNatomy (TN; Dentsply Sirona, Maillefer, Ballaigues, Switzerland) rotary files were selected. With the use of a high-speed diamond bur, they were taken out of their sealed boxes and sequentially numbered on the handles. A stereomicroscope (SMZ 1500, Nikon Inc. and Melville, NY) was then used to examine the files at magnifications ranging from 20X to 100X. Initially an access cavity was made by using an endodontic access diamond friction grip bur (size 1) (Dentsply/Maillefer, Ballaigues, Switzerland) and straight-line access for the mesial wall was performed by using the Endo-Z stainless steel bur (Dentsply/ Maillefer, Ballaigues, Switzerland). Working length was determined by using a size 10 K-type file (Dentsply/ Maillefer, Ballaigues, Switzerland) and Electronic Apex locator (Root ZX II-J, Morita, Tokyo, Japan). Additionally, this study only examined root canals in teeth that had an initial apical size of #10 or #15 K-file (Mani, Japan). Both the file systems were used according to the manufacturer's instructions. Each file was used to prepare 2 mesial canals in each tooth and evaluated after each use. They are sterilized in between by autoclave sterilization.

The groups are further subdivided based on the temperature of NaOCl used as the irrigants.

[1] Group 1 OC - Room Temperature Sodium Hypochlorite.

[2] Group 2 OCp - Preheated Sodium Hypochlorite.

[3] Group 3 TN - Room Temperature Sodium Hypochlorite.

[4] Group 4 TNp - Preheated Sodium Hypochlorite.

For NaOCl, heating double boiler method with water bath was used [8] [2]. The temperature was maintained with laboratory thermometer at 60°C. The NaOCl thus formed was used according to the groups formed. The files were rotated in each tooth for decided 5 minutes with irrigation with 5.25% sodium hypochlorite. The files were inspected at a distance of 3-5 mm from the tip for the SEM measurement of surface roughness. Stereomicroscope analysis was done on the files for Unwinding and Distortion.

## Stereomicroscopic analysis:

According to Sotokawa (1988), Roane and Sabala (1984), Idomeo Bonetti Filho *et al.* and other authors, there are several classifications of various sorts of faults (1998) [8]. Although there are already a few classes, a new classification was created to help professionals- visually and microscopically assess torsional fatigue and make the study more relevant. The benefit of this categorization is that it also indicates how serious the flaw or harm is. According to severity of the flaws, there are two categories in this categorization for torsional fatigue challenges (Figure 1, 2, 3 and 4 - see PDF).

## Defects that suggest little damage:

[1] Bent instrument or deformed tip.

[2] Stretched or straightened twist shape.

[3] Dented cutting edge.

## Errors that suggest extensive damage:

[1] Alteration in length (mm),

[2] Partial reverse twisting and

[3] Instrument fracture

Following the aforesaid classification, scores were assigned based on the presence or absence of distortion after inspecting each file under a microscope and visually after each use.

## SEM analysis:

The instruments were then positioned conventionally on a stub for SEM investigation. Quanta FEG 400 was used to scan the samples (FEI, USA). The photos of the active portion (region of interest), the 4 mm distal end, were processed using the common 120, 250 and 1000 magnifications. Two calibrated examiners who examined all SEM pictures on a computer screen recorded whether and what kind of deformations occurred in the files. ED's measurements were made and these showed that all instruments' chemical compositions had changed.

## Statistical analysis:

The frequency distribution of observed deformation between the 1^st^ use and 2^nd^ clinical use in four groups has been checked. We used chi-square test and logistic regression model (when no Xero observation found). A p-value of 0.05 has been considered as significant. All the statistical comparison was made with STATA (version 15).

## Results:

Table 1 details the types of deformations observed in the OC group after the first and second clinical uses. After the first use involving 3 canals, 2 (20%) of instruments showed deformation or distortion, none exhibited unwinding or tip deformation, 2 (20%) had blade disruption and there were no cases of surface pitting or micro-cracks. Following the second use involving 6 canals, deformation or distortion increased to 5 (50%), unwinding appeared in 1 (10%) of the instruments, there was still no tip deformation, blade disruption rose to 4 (40%), surface pitting was observed in 2 (20%) and micro-cracks appeared in 1 (10%) of the instruments. (Figure 5, 6 and 7 - see PDF) This indicates an increase in various forms of instrument wear and damage with repeated use, similar to the OCp group. To estimate the association between the first and second uses, we applied both the chi-square test and a binary logistic regression model. However, none of the deformations exhibited any significant changes. Table 2 shows the types of deformations observed in the OCp group after the first and second clinical uses. Following the first use involving 3 canals, 4 (40%) of instruments showed deformation or distortion, none exhibited unwinding or tip deformation, 2(20%) had blade disruption, 1 (10%) had micro-cracks and none showed surface pitting. After the second use involving 6 canals, deformation or distortion increased to 6(60%), unwinding and tip deformation both appeared in 1 (10%) of the instruments, blade disruption rose to 3 (30%), surface pitting was observed in 2 (20%) and micro-cracks were seen in 3 (30%) of the instruments. This data indicates a general increase in various forms of instrument wear and damage with repeated clinical use. To estimate the association between the 1st and 2nd use we used the chi-square and binary logistic regression model but none of the deformation showed any significant changes. Table 3 details the types of deformations observed in the TP group after the first and second clinical uses. After the first use involving 3 canals, 10% of instruments showed deformation or distortion, and none exhibited unwinding, tip deformation, surface pitting, or micro-cracks. Additionally, 10% had blade disruption. Following the second use involving 6 canals, deformation or distortion increased to 40%, there were still no instances of unwinding or tip deformation, blade disruption rose to 20%, surface pitting appeared in 10% of the instruments and micro-cracks were observed in 20% of the instruments. This data shows an increase in forms of instrument wear and damage with repeated clinical use (Figure 8, 9, 10 - see PDF). Similarly, with the other groups TP also not demonstrated any significant difference between the 1st and 2nd use. Table 4 details the types of deformations observed in the TNp group after the first and second clinical uses. Following the first use involving 3 canals, 20% of instruments showed deformation or distortion, none exhibited unwinding, tip deformation, surface pitting, or micro-cracks and 20% had blade disruption. After the second use involving 6 canals, deformation or distortion increased to 50%, unwinding appeared in 10% of the instruments, there was still no tip deformation, blade disruption rose to 30%, surface pitting was observed in 30% and micro-cracks were seen in 20% of the instruments. This indicates a notable increase in instrument wear with repeated clinical use.

## Discussion:

Griffith law states that after a metal reaches its fatigue strength, the NiTi alloy acts as brittle item 1 Alpati *et al.* noted the apparent enlargement of the machining crack as a result of the buildup of dentinal debris [2, 3, 4- 5]. Micro-crack initiation is significantly influenced by the machining grooves (A.L. Gloanec) [3]. It's crucial to assess how the topography of endodontic files' metal surfaces affects how resistant they are to wear. In order to determine the topography, a number of investigations were conducted using Stereomicroscope and SEM [9-10]. For changes which occur in the surface such as micro-cracks and pitting, this cannot be observed in a stereomicroscope. SEM analysis was chosen as it allows for the evaluation of morphological traits and NiTi file deformation under high magnification [9, 11, 12]. In this study, we compared two different file systems. A single NiTi rotary device for shaping root canals called One Curve (Micro Mega) was released in 2017 [3]. The One Curve file's adjustable cross section enhances centering and cutting effectiveness. One Curve file reportedly has a 2.4 times greater cyclic fatigue resistance than the One Shape system [3]. M wire technology is used to create TruNatomy [9]. M-wire has smaller grains than traditional NiTi wires. Ye *et al.* [5] suggested that M-100 Wire's nm martensite grains helped it to have better torque resilience and resistance to wear. Lower grain sizes of the alloy can increase yield strength by reducing crack initiation along grain boundaries brought on by locally concentrated stress [3]. It was found that in stereomicroscopy, unwinding and surface changes of One Curve were more as compared to TruNatomy. Each file from all 4 groups was further rotated in artificial curved canals until they fractured. It was noted that group 2 files were the first files to fracture followed by group 4, group 1 and group 3. The cyclic fatigue resistance of TruNatomy was significantly longer than that of One Curve. After each use, the files underwent certain metallurgical modifications. After both uses, there were blunt cutting edges visible, possibly due to reduction in strength of file under cycles and a concentration of strain in these regions [1]. Cutting edge disruption and fracture were observed in certain files; this might be explained by the stress distribution across the micro-cracks [4]. Parallel micro-cracks began to appear on the edges at the files' tips after the initial use, which indicates how much pressure was applied to the tip [10]. Pitting corrosion requires the presence of two metals acting as the anode and cathode in the presence of a media or electrolyte (NaOCl) that also needs to be present [11]. Mechanical stresses cause the passive TiO2 to degrade and the resulting Ti may aid in galvanic corrosion. Using preheated NaOCl caused more pitting corrosion in the files in both the groups. Though there was not much statistically significant difference, in the current investigation, it was discovered that TruNatomy outperformed the other One Curve, heat-treated files in terms of corrosion resistance to NaOCl. This result suggests that the file's sensitivity to NaOCl corrosion is not always increased by heat treatment alone. A disparity in the file's susceptibility to NaOCl corrosion may result from a variety of manufacturing parameters, including the file's cross-section geometry, design and surface treatment used after carving. Moreover, the file's greater resistance to NaOCl corrosion may be due to the visible blue titanium oxide surface layer [1, 11-12].

The EDS of both the files compared when used with preheated NaOCl resulted in presence of oxygen in both the files. Though it was noted that oxygen were more pronounced in the ONE CURVE group 2. This could be again because of the surface treatment of TN. In contrast to other investigations, the findings of this study demonstrated a low incidence of topographic alterations, absence of fracture and a robust resistance against cyclic fatigue [10, 9]. Many elements, including NiTi alloys and the M-wire technology, which gives the files more flexibility, as well as the off-center rectangular cross-sectional shape, might account for this disparity. TN performed better as compared to OC. Though no other studies have compared these two files so further studies need to evaluate the same results. There is an increased mechanical damage seen to the OC system after the second use, but the data does not show significant differences. To ascertain the actual risk of damage more research with larger sample sizes could be necessary.

## Conclusion:

Surface roughness (depth of grooves) influenced the fracture resistance of the examined endodontic instruments. TN showed less surface deformations as compared to OC, though the results are statistically insignificant. Thus, both files can be used safely to instrument in up to 4 curved canals. Irrigants such as NaOCl, in preheated form affects the surface topography and the fatigue resistance of the files.

## Figures and Tables

**Table 1:** The kinds of deformations after first and second use of surface modifications after clinical use in OC group

**OC**	**1^st^ use (3 canals)**	**After 2^nd^ use (6 canals)**	**OR(95%CI)**	**p-value**
Deformation/Distortion				
Presence	2(20.0%)	5(50.0%)	4.00(0.55, 29.1)	0.171*
Absence	8(80.0%)	5(50.0%)		
Unwinding				
Presence	0	1(10.0%)		0.305#
Absence	10(100.0%)	9(90.0%)		
Tip Deformation				
Presence	0	0		--
Absence	10	10		
Blade disruption				
Presence	2(20.0%)	4(40.0%)	2.67(0.36, 19.7)	0.337*
Absence	8(80.0%)	6(60.0%)		
Surface pitting				
Presence	0	2(20.0%)		0.136#
Absence	10(100%)	80(80.0%)		
Micro-cracks				
Presence	0	1(10.0%)		0.305#
Absence	10(100%)	9(90.0%)		
*P-values were estimated by
using logistic regression model.
#P-values was estimated by
chi-square test.

**Table 2:** Types of deformations after 1^st^ and 2^nd^ use of surface changes after clinical use in OCp group

**OCp**	**1^st^ use (3 canals)**	**After 2^nd^ use (6 canals)**	**OR(95%CI)**	**p-value**
Deformation/Distortion				
Presence	4(40.0%)	6(60.0%)	2.25(0.37, 13.5)	0.374*
Absence	6(60.0%)	4(40.0%)		
Unwinding				
Presence	0	1(10.0%)		0.305#
Absence	10(100.0%)	9(90.0%)		
Tip Deformation				
Presence	0	1(10.0%)		0.305#
Absence	10(100.0%)	9(90.0%)		
Blade disruption				
Presence	2(20.0%)	3(30.0%)	1.71(0.22, 13.4)	0.608*
Absence	8(80.0%)	7(70.0%)		
Surface pitting				
Presence	0	2(20.0%)		0.136#
Absence	10(100%)	80(80.0%)		
Micro-cracks				
Presence	1	3(30.0%)	3.86(0.33, 45.6)	0.284#
Absence	9(90%)	7(70.0%)		
*P-values were estimated by
using logistic regression model.
#P-values was estimated by
chi-square test.

**Table 3:** Types of deformations after 1^st^ and 2^nd^ use of surface changes after clinical use in TP group

**TP**	**1^st^ use (3 canals)**	**After 2^nd^ use (6 canals)**	**OR(95%CI)**	**p-value**
Deformation/Distortion				
Presence	1(10.0%)	4(40.0%)	6.00(0.53, 67.6)	0.147*
Absence	9(90.0%)	6(60.0%)		
Unwinding				
Presence	0	0		
Absence	10(100.0%)	10(90.0%)		
Tip Deformation				
Presence	0	0		
Absence	10(100.0%)	10(90.0%)		
Blade disruption				
Presence	1(10.0%)	2(20.0%)	2.25(0.17, 29.8)	0.538*
Absence	9(90.0%)	8(80.0%)		
Surface pitting				
Presence	0	1(10.0%)		0.305#
Absence	10(100%)	9(80.0%)		
Micro-cracks				
Presence	0	2(20.0%)		0.136#
Absence	10(100.0%)	8(80.0%)		
*P-values were estimated by
using logistic regression model.
#P-values was estimated by
chi-square test.

**Table 4:** Types of deformations after 1^st^ and 2^nd^ use of surface changes after clinical use in TNp group

**TNp**	**1^st^ use (3 canals)**	**After 2^nd^ use (6 canals)**	**OR(95%CI)**	**p-value**
Deformation/Distortion				
Presence	2(20.0%)	5(50.0%)	4.00(0.55, 29.1)	0.171*
Absence	8(80.0%)	5(50.0%)		
Unwinding				
Presence	0	1(10.0%)		0.305#
Absence	10(100.0%)	9(90.0%)		
Tip Deformation				
Presence	0	0		
Absence	10(100.0%)	10(100.0%)		
Blade disruption				
Presence	2(20.0%)	3(30.0%)	1.71(0.22, 13.4)	0.608#
Absence	8(80.0%)	7(70.0%)		
Surface pitting				
Presence	0	3(30.0%)		0.060#
Absence	10(100%)	7(70.0%)		
Micro-cracks				
Presence	0	2(20.0%)		0.136#
Absence	10(100.0%)	8(80.0%)		
*P-values were estimated by
using logistic regression model.
#P-values was estimated by
chi-square test.
